# Long Working Hours, Work-life Imbalance, and Poor Mental Health: A Cross-sectional Mediation Analysis Based on the Sixth Korean Working Conditions Survey, 2020–2021

**DOI:** 10.2188/jea.JE20230302

**Published:** 2024-11-05

**Authors:** Seong-Uk Baek, Yu-Min Lee, Jin-Ha Yoon, Jong-Uk Won

**Affiliations:** 1 Department of Occupational and Environmental Medicine, Severance Hospital, Yonsei University College of Medicine, Seoul, Korea; 2 The Institute for Occupational Health, Yonsei University College of Medicine, Seoul, Korea; 3 Graduate School, Yonsei University College of Medicine, Seoul, Korea; 4 Graduate School of Public Health, Yonsei University College of Medicine, Seoul, Korea; 5 Department of Preventive Medicine, Yonsei University College of Medicine, Seoul, Korea

**Keywords:** overwork, well-being, work-family conflict, work-life spillover, depressive symptoms

## Abstract

**Background:**

There has been growing concern about the negative mental health impact of long working hours and overwork. Our study examined how work-life imbalance (WLI) could be a mediator between working hours and poor mental well-being.

**Methods:**

We included 34,968 individuals from a nationwide cross-sectional survey in Korea. Self-reported working hours per week were collected, and mental health was assessed using the World Health Organization (WHO)-5 Well-Being Index. Counterfactual-based mediation models were employed to disentangle the total effects into a direct effect (work hour – poor mental health) and an indirect effect (work hour – WLI – poor mental health).

**Results:**

Out of 34,968 participants, 52.6% worked 35–40 hours/week, 20.0% worked 41–48 hours/week, 11.7% worked 49–54 hours/week, and 15.6% worked ≥55 hours/week. The odds ratios (ORs) of the total impact of working hours on poor mental health were 1.08 (95% confidence interval [CI], 1.01–1.16) for 41–48 hours/week, 1.28 (95% CI, 1.17–1.39) for 49–54 hours/week, and 1.60 (95% CI, 1.48–1.74) for ≥55 hours/week in comparison to 35–40 hours/week. The ORs of the indirect effects were 1.04 (95% CI, 1.03–1.05) for 41–48 hours/week, 1.08 (95% CI, 1.07–1.09) for 49–54 hours/week, and 1.14 (95% CI, 1.12–1.16) for ≥55 hours/week, accounting for 51%, 31%, and 28% of the total effects, respectively.

**Conclusion:**

Our findings suggest that WLI can partially mediate the association of long working hours with mental health deterioration. Policy efforts are required to mitigate the adverse mental health effects of overwork.

## INTRODUCTION

The adverse health effects of exposure to long working hours and overwork have recently attracted considerable social and academic interest. According to studies by the World Health Organization (WHO) and the International Labor Organization (ILO), long working hours, defined as working 55 hours per week or more, can cause ischemic heart disease and stroke.^1^^,^^2^ Globally, exposure to long working hours are estimated to be the cause of approximately 745,000 deaths annually, and the burden of long working hours is disproportionally distributed among workers in the Western-Pacific regions, men, and older workers.^3^ Despite a gradual reduction in working hours in South Korea over the past few decades, it remains one of the nations with the highest annual working hours.^4^^,^^5^ Specifically, South Korea adheres to a 52-hour workweek policy, contrasting with Europe and the ILO, where regulations govern working hours that exceed 48 hours per week.^4^^,^^5^ Furthermore, a collectivist organizational culture prioritizing commitment to work over work-family balance is more prevalent compared to Europe.^6^ In recent years, working hours policies in Korean society have sparked debates, drawing the attention of not only public health researchers but also policymakers and the broader workforce.^7^

In addition to physical diseases, the existing literature has explored the effects of long work hours and overwork on mental well-being. A meta-analyses conducted by WHO/ILO and Watanabe et al have maintained that there is insufficient evidence to support the causal effect of long hours of working on the onset of psychiatric disorders.^8^^,^^9^ Nevertheless, other studies have indicated a close association of long working hours with psychological well-being, including depressive symptoms.^10^^,^^11^ For instance, a meta-analysis by Virtanen et al has suggested that long working hours that were defined as working ≥55 hours per week are significantly related to an increased risk of depressive symptoms.^10^ Several cross-sectional and longitudinal studies have also found a direct association of long working hours with the risk of poor mental health, such as depressive symptoms or suicidal ideation.^12^^–^^16^

The effect of extended work hours on the mental health conditions of workers has been assumed to be mediated through multiple mechanisms, including disturbance of work-life balance, emotional distress, exhaustion, unhealthy lifestyle behaviors, and physiological changes.^8^ Studies have maintained that long working hours are contributors to work-life imbalance (WLI) among workers.^17^^–^^19^ For instance, individuals experiencing long working hours may face difficulties in allocating time to socialize with their family and friends.^20^^–^^22^ Furthermore, long working hours may trigger persistent rumination about work matters, even during personal time, leading to adverse consequences for mental well-being, such as burnout symptoms.^23^ Previous studies have documented the detrimental effects of WLI on mental well-being, such as depressive symptoms, fatigue, and sleep problems.^24^^–^^26^ Consequently, based on existing literature, it can be hypothesized that an increase in WLI could serve as a mediator in the association between long working hours and poor mental well-being.

Previous studies have suggested that WLI may play a mediating role in the associations between long commute time and anxiety or sleep disturbance,^27^ between work-time control and depressive symptoms,^28^ and between working from home and depression or anxiety symptoms.^29^ However, to the best of our knowledge, the mediating role of WLI on the relationship between long working hours and poor mental well-being has not been fully investigated in the existing literature. Hence, in this study, we explored two main research questions:

(i) Is there a positive association of long working hours with poor mental health?
(ii) Does WLI mediate the association of long working hours with poor mental health?

## METHODS

### Study sample

The research participants were selected from the sixth survey wave of the Korean Working Conditions Survey (KWCS), a nationwide survey conducted carried out triennially by the Occupational Safety and Health Research Institute.^30^^,^^31^ The KWCS employs a systemic sampling method, in which enumeration districts are selected as the primary sampling units. Therefore, the KWCS consists of nationally representative worker sample in Korea.^31^ The sixth KWCS was conducted from October 2020 to April 2021.

Initially, 50,538 workers were included in the 2020–2021 survey year of the KWCS. We excluded individuals aged ≥65 years (*n* = 8,401) as this is the retirement age. Next, we included either employees or the self-employed and excluded unpaid family workers (*n* = 1,015). Consistent with previous WHO/ILO studies, individuals working <35 hours/week were excluded. The study sample comprised 34,968 workers, with 273 individuals without information about working hours excluded during this process (eFigure 1). The rationale for excluding those workers was that working 35–40 hours per week was considered a minimum risk exposure and was used as a reference point in prior meta-analyses^8^^,^^32^ and other studies that examined the mental effects of long work hours.^10^^,^^33^

### Ethics statement

Raw data for the KWCS are publicly available at https://www.kosha.or.kr. This study was approved by the Institutional Review Board of the Yonsei Health System (No. 4-2022-1507).

### Variables

#### Working hours

Respondents were asked the following question: “On average, what is the weekly working hours for your work at your workplace?” We pre-established the categorization of working hours by adopting the cut-off values employed in prior studies examining the mental health effects of long working hours, including meta-analyses conducted by WHO and the ILO.^8^^,^^10^^,^^34^ working hours per week were classified as “35–40 hours,” “41–48 hours,” “49–54 hours,” and “≥55 hours.” Working 35–40 hours per week (standard working hours) was defined as the reference group.^8^^,^^34^

#### Work-life imbalance

WLI was evaluated using five items derived from the work-life balance scale in the EWCS (eTable 1).^35^^–^^37^ Examples of questions include “How frequently in the past 12 months have you experienced your job preventing you from allocating the time you desired for your family?” or “How often in the last 12 months have your family responsibilities hindered you from dedicating the time necessary to your job?” Participants provided responses on a 5-point Likert scale, ranging from 1 (“Never”) to 5 (“Always”) for each question. The total score was obtained by summing the scores for each item. A higher score indicated a higher level of WLI. Cronbach’s alpha was 0.84. The results of the factor analysis (eTable 2 and eTable 3) indicated that the WLI scale was unidimensional. The summed score of WLI items that ranged from 5 to 25, was treated as a continuous scale.

#### Outcome: mental health

Poor mental health was assessed based on the WHO-5 Well-being Index (WHO-5 index), which is a widely used tool for screening depressive symptoms.^38^ The WHO-5 index consists of five items regarding the vigor and emotions in the last 2 weeks. An example item is “Have you felt calm and relaxed during the past 2 weeks?” Individuals responded on a score ranging from 0 (“At no time”) to 5 (“All of the time”). The overall score was obtained by summing each individual score and then multiplying it by four, with a higher score indicating a better sense of well-being. Following a previous study,^38^ a total score of less than 50 was considered indicative of poor mental health.

#### Covariates

Several covariates were adjusted: gender, age group (<40, 40–49, 50–59, 60–65 years), education level (Middle school or below, High school, College or above), monthly income (“<₩2,000,000” “₩2,000,000–2,990,000” “₩3,000,000–3,990,000” “≥₩4,000,000”), marital status (Married, Unmarried or others), number of household members (1, 2, 3–4, ≥5), type of employment (Employees, Self-employed workers), and occupation (“Blue collars, Service/sales workers, White collars).

### Statistical analysis

First, the characteristics according to work hours were examined. The prevalence of poor mental health was explored for its relation to study variables.

Second, prior to conducting the mediation analysis, a preliminary investigation was undertaken to examine the potential associations between the variables in the two indirect paths (eMaterial 1). This examination involved the linear and logistic regression models. The following relationship were investigated:

(i) The relationship between long working hours and WLI (working hours → WLI score)
(ii) The relationship between WLI and poor mental health (WLI score → poor mental health)

**Figure 1.**
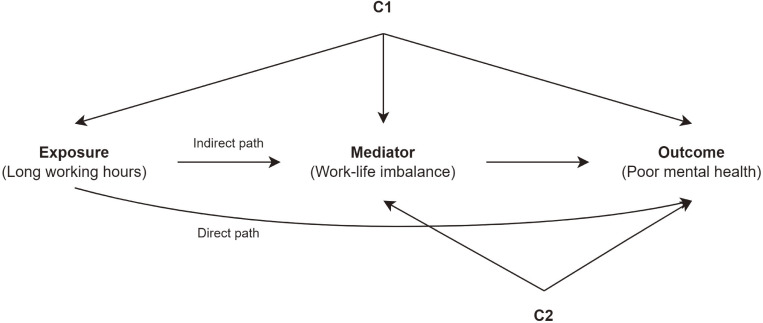
Diagram illustrating the mediating effect of work-life imbalance in the relationship between long work hours and poor mental health. The identified confounding factor C1 encompasses gender, age, education, marital status, and the number of household members, while confounding factor C2 includes monthly income, occupation, and employment type.

Mediation analyses were carried out with the premise of considering two primary pathways connecting long work hours to diminished mental well-being, as shown in Figure 1. The first path is the direct path, in which long work hours are related to the outcome (poor mental health), regardless of WLI. The second path is the indirect path, where long work hours are related to the outcome (poor mental health) because long working hours are related to an increased level of WLI. We performed a counterfactual-based mediation analysis.^39^ This method enables the decomposition of the total effect into indirect and direct effects in a logit model based on the potential outcomes framework, allowing any distribution of the mediating variable (see eMaterial 2). The effect size was presented as odds ratios (ORs) with 95% confidence intervals (CIs). To estimate CIs, we utilized 1,000 bootstrap resamplings. The proportion mediated was calculated by dividing the indirect effects by the total effect. Data preparation and descriptive analyses were conducted using the R software (version 4.2.3; R Foundation for Statistical Computing, Vienna, Austria). Regression and mediation analyses were conducted using Stata (version 18.0; StataCorp LLC, College Station, TX, USA). Mediation analysis was conducted using “*ldecomp*” package.^39^

#### Missing values and sensitivity analysis

The proportion of missing values is presented in Table 1. For the regression and mediation analyses, missing values were handled through multiple imputations, assuming missing at random (MAR). Twenty datasets were generated, and estimates were combined based on Rubin’s rule using “*mi estimate*” command in Stata.

**Table 1.** Distribution of characteristics of study participants

Characteristics	Overall	Working hours per week				

		35–40 hours	41–48 hours	49–54 hours	≥55 hours	*P* value^b^

	*N* = 34,968	*N* = 18,397	*N* = 7,013	*N* = 4,099	*N* = 5,459	
WLI						
Mean (standard deviation)	9.8 (3.6)	9.3 (3.5)	9.9 (3.6)	10.4 (3.7)	11.1 (3.7)	<0.001
Missing values	1,985	994	371	253	357	
Gender						
Men	17,733 (50.7)	9,175 (49.9)	3,405 (48.6)	2,252 (54.9)	2,901 (53.1)	<0.001
Women	17,235 (49.3)	9,222 (50.1)	3,608 (51.4)	1,847 (45.1)	2,558 (46.9)	
Age, years						
<40	11,501 (32.9)	6,867 (37.3)	2,232 (31.8)	1,193 (29.1)	1,209 (22.1)	<0.001
40–49	9,481 (27.1)	5,274 (28.7)	1,815 (25.9)	1,106 (27)	1,286 (23.6)	
50–59	10,335 (29.6)	4,877 (26.5)	2,173 (31.0)	1,272 (31)	2,013 (36.9)	
≥60	3,651 (10.4)	1,379 (7.5)	793 (11.3)	528 (12.9)	951 (17.4)	
Education						
Middle school or below	1,567 (4.5)	606 (3.3)	351 (5)	222 (5.4)	388 (7.1)	<0.001
High school	13,227 (37.9)	5,359 (29.2)	2,952 (42.1)	1,890 (46.2)	3,026 (55.5)	
College or above	20,134 (57.6)	12,414 (67.5)	3,703 (52.9)	1,981 (48.4)	2,036 (37.4)	
Missing values	40	18	7	6	9	
Marital status						
Married	22,006 (62.9)	11,488 (62.4)	4,344 (61.9)	2,592 (63.2)	3,582 (65.6)	<0.001
Unmarried or others	12,962 (37.1)	6,909 (37.6)	2,669 (38.1)	1,507 (36.8)	1,877 (34.4)	
Number of household members						
1	6,940 (19.8)	3,526 (19.2)	1,450 (20.7)	847 (20.7)	1,117 (20.5)	<0.001
2	8,834 (25.3)	4,277 (23.2)	1,945 (27.7)	1,048 (25.6)	1,564 (28.6)	
3–4	8,968 (25.6)	4,833 (26.3)	1,771 (25.3)	1,101 (26.9)	1,263 (23.1)	
≥5	10,226 (29.2)	5,761 (31.3)	1,847 (26.3)	1,103 (26.9)	1,515 (27.8)	
Monthly income (₩)						
<2,000,000	5,637 (16.9)	3,107 (17.7)	1,278 (18.9)	479 (12.3)	773 (15.0)	<0.001
2,000,000–2,990,000	12,510 (37.6)	6,406 (36.6)	2,720 (40.3)	1,548 (39.8)	1,836 (35.6)	
3,000,000–3,990,000	8,492 (25.5)	4,343 (24.8)	1,634 (24.2)	1,093 (28.1)	1,422 (27.6)	
≥4,000,000	6,670 (20.0)	3,662 (20.9)	1,113 (16.5)	773 (19.9)	1,122 (21.8)	
Missing values	1,659	879	268	206	306	
Occupation						
White collar	14,616 (41.8)	10,617 (57.7)	2,438 (34.8)	1,005 (24.5)	556 (10.2)	<0.001
Service and sales worker	10,747 (30.7)	3,358 (18.3)	2,379 (33.9)	1,756 (42.8)	3,254 (59.6)	
Blue collar	9,605 (27.5)	4,422 (24.0)	2,196 (31.3)	1,338 (32.6)	1,649 (30.2)	
Employment type						
Employee	24,848 (71.1)	16,336 (88.8)	4,862 (69.3)	2,220 (54.2)	1,430 (26.2)	<0.001
Self-employed	10,120 (28.9)	2,061 (11.2)	2,151 (30.7)	1,879 (45.8)	4,029 (73.8)	
Poor mental health						
No	27,529 (78.8)	14,947 (81.4)	5,549 (79.2)	3,129 (76.4)	3,904 (71.6)	<0.001
Yes	7,385 (21.2)	3,414 (18.6)	1,456 (20.8)	966 (23.6)	1,549 (28.4)	
Missing values	54	36	8	4	6	

WLI, Work-life imbalance.

^a^2,000,000 KRW = 1,495 USD = 222,372 JPY; 3,000,000 KRW = 2,243 USD = 333,558 JPY; 4,000,000 KRW = 2,991 USD = 444,744 JPY.

^b^Chi-squared test for categorical variables and ANOVA for continuous variables.

Values are presented as *N* (%) for categorical variables and mean (standard deviation) for continuous variables.

Several sensitivity analyses were performed. First, we repeated our mediation analysis using complete cases without any missing values (*N* = 31,418). Second, we applied an additional categorization for working hours with the same intervals and adhering to the 52-hour working policy in Korea: (i) 35–40 hours, (ii) 41–46 hours, (iii) 47–52 hours, and (iv) >52 hours per week. Third, we performed a gender-stratified analysis to investigate how the association between long working hours and poor mental health differs by gender (men vs women). Fourth, we performed stratified analyses by occupation types to investigate how job-control might affect the workhour-WFI-mental health relationship (white-collar vs non-white-collar workers). Fifth, we conducted stratified analysis by the household composition (single vs non-single households) to consider the heterogeneity of the burden of household chores by household composition.

## RESULTS

Out of 34,968 participants, 18,397 (52.6%) worked 35–40 hours per week, 7,013 (20.0%) worked 41–48 hours per week, 4,099 (11.7%) worked 49–54 hours per week, and 5,459 (15.6%) worked ≥55 hours per week (Table 1). Individuals who worked long hours (≥55 hours per week) were more likely to be men, aged 50 years or older, had lower educational attainment, worked in non-white collar occupations, and were self-employed workers.

The prevalence of poor mental health was 18.6% among those working 35–40 hours per week, 20.8% among those working 41–48 hours per week, 23.6% among those working 48–54 hours per week, and 28.4% among those working 55 hours or more per week (Table 2). The prevalence of poor mental health was also higher among workers with low education level or low income level, service/sales workers, blue-collar workers, and the self-employed workers.

**Table 2.** Prevalence of poor mental health according to study variables

Characteristics	Poor mental health		

	Yes	No	*P* value^b^

	*N* = 7,385	*N* = 27,529	
Working hours per week			
35–40 hours	3,414 (18.6)	14,947 (81.4)	<0.001
41–48 hours	1,456 (20.8)	5,549 (79.2)	
49–54 hours	966 (23.6)	3,129 (76.4)	
≥55 hours	1,549 (28.4)	3,904 (71.6)	
Gender			
Men	3,838 (21.7)	13,865 (78.3)	0.014
Women	3,547 (20.6)	13,664 (79.4)	
Age, years			
<40	2,035 (17.7)	9,442 (82.3)	<0.001
40–49	2,004 (21.2)	7,459 (78.8)	
50–59	2,405 (23.3)	7,921 (76.7)	
≥60	941 (25.8)	2,707 (74.2)	
Education			
Middle school or below	492 (31.4)	1,074 (68.6)	<0.001
High school	3,234 (24.5)	9,973 (75.5)	
College or above	3,645 (18.1)	16,457 (81.9)	
Missing values	14	25	
Marital status			
Married	4,671 (21.3)	17,304 (78.7)	0.500
Unmarried or others	2,714 (21.0)	10,225 (79)	
Number of household members			
1	1,470 (21.2)	5,459 (78.8)	0.600
2	1,908 (21.6)	6,911 (78.4)	
3–4	1,884 (21.0)	7,074 (79.0)	
≥5	2,123 (20.8)	8,085 (79.2)	
Monthly income (₩)^a^			
<2,000,000	1,524 (27.1)	4,107 (72.9)	<0.001
2,000,000–2,990,000	2,591 (20.7)	9,904 (79.3)	
3,000,000–3,990,000	1,624 (19.1)	6,858 (80.9)	
≥4,000,000	1,143 (17.2)	5,517 (82.8)	
Missing values	503	1,143	
Occupation			
White collar	2,641 (18.1)	11,946 (81.9)	<0.001
Service and sales worker	2,326 (21.7)	8,413 (78.3)	
Blue collar	2,418 (25.2)	7,170 (74.8)	
Employment type			
Employee	4,906 (19.8)	19,898 (80.2)	<0.001
Self-employed	2,479 (24.5)	7,631 (75.5)	

^a^2,000,000 KRW = 1,495 USD = 222,372 JPY; 3,000,000 KRW = 2,243 USD = 333,558 JPY; 4,000,000 KRW = 2,991 USD = 444,744 JPY.

^b^Chi-squared test for categorical variables and ANOVA for continuous variables.

Values are presented as *N* (row %). 54 observations with missing mental health status data were excluded from this table.

Table 3 demonstrates the association of long working hours with WLI (model A) and of WLI with poor mental health (model B). The β of the association between working hours per week and the WLI score was 0.40 (95% CI, 0.30–0.50) for 41–48 hours, 0.77 (95% CI, 0.64–0.89) for 49–54 hours, and 1.23 (95% CI, 1.10–1.36) for ≥55 hours compared to 35–40 hours, in the fully adjusted model. Additionally, a 1-point increase in the WLI score was positively associated with the OR for having poor mental health (OR 1.08; 95% CI, 1.07–1.09) (model B).

**Table 3.** Association between long working hours and WLI and between WLI and poor mental health (*N* = 34,968)

Path	Model A (Dependent variable: WLI)				Model B (Dependent variable: poor mental health)			

	Crude model		Adjusted model		Crude model		Adjusted model	

	*β*	95% CI	*β*	95% CI	OR	95% CI	OR	95% CI
**Working hours**								
35–40 hours	0.00	Reference	0.00	Reference				
41–48 hours	0.55	0.45–0.65	0.55	0.45–0.65				
49–54 hours	1.06	0.94–1.19	1.06	0.94–1.19				
≥55 hours	1.81	1.70–1.92	1.81	1.70–1.92				
**WLI**								
Continuous variable (range 5–25)					1.08	1.07–1.09	1.08	1.07–1.09

CI, confidence interval; OR, odds ratio; WLI, work-life imbalance.

The adjusted model controlled for gender, age, education, marital status, number of household members, monthly income, occupation, and employment type.

Table 4 shows the total, indirect, and direct effects of long working hours on poor mental health. The OR of the total effect of long working hours per week on poor mental health was 1.08 (95% CI, 1.01–1.16) for 41–48 hours, 1.28 (95% CI, 1.17–1.39) for 49–54 hours, and 1.60 (95% CI, 1.48–1.74) for ≥55 hours compared to 35–40 hours. The indirect effect of long working hours per week on poor mental health mediated through WLI was 1.04 (95% CI, 1.03–1.05) for 41–48 hours, 1.08 (95% CI, 1.07–1.09) for 49–54 hours, and 1.14 (95% CI, 1.12–1.16) for ≥55 hours, accounting for 50.8%, 31.3%, and 27.6% of the total effect, respectively.

**Table 4.** Total, direct, and indirect effects of long working hours on poor mental health (*N* = 34,968)

	Total effect		Indirect effect		Direct effect		PM
			
	OR	95% CI	OR	95% CI	OR	95% CI	%
**Working hours per week**							
35–40 hours	1.00	reference	1.00	reference	1.00	reference	
41–48 hours	1.08	1.01–1.16	1.04	1.03–1.05	1.04	0.97–1.11	50.8
49–54 hours	1.28	1.17–1.39	1.08	1.07–1.09	1.18	1.09–1.29	31.3
≥55 hours	1.60	1.48–1.74	1.14	1.12–1.16	1.41	1.30–1.53	27.6

CI, confidence interval; OR, odds ratio; PM, proportion mediated.

The model controlled for gender, age, education, marital status, number of household members, monthly income, occupation, and employment type.

eTable 4 shows the results of the complete-case analysis (*N* = 31,418). Complete-case analysis also confirmed the mediating role of WLI on the association between long working hours and poor mental health. Additional analysis with a different cut-off for working hours also shows the significant indirect effect of long working hours on poor mental health mediated through WLI (eTable 5). The indirect impacts of long working hours were found to be comparable for both men and women (eTable 6). The mediating impact of long working hours on poor mental health through WLI was observed in both white-collar workers and non-white workers (eTable 7). Lastly, eTable 8 indicates that the mediating effect of long working hours on poor mental health was observed to be similar between workers in single household and those in non-single household.

## DISCUSSION

The primary findings of our study are twofold. First, working more than 40 hours per week was positively associated with poor mental health. Second, our study revealed that WLI partially mediated the relationship between the exposure to long work hours and poor mental health. Specifically, long working hours may lead to a heightened level of WLI among workers, consequently contributing to an elevated OR for experiencing poor mental health. This relationship follows a dose-response pattern, in which longer working hours are related to higher ORs for both the total and indirect effects on poor mental health. WLI accounted for approximately 27.6–50.8% of the elevated likelihood for the poor mental health observed in those experiencing long working hours.

Our findings are consistent with previous investigations showing a positive association of long working hours with poor mental health.^12^^–^^16^ Additionally, previous studies employing the WHO-5 index for outcomes consistently demonstrated a positive association between long working hours and poor mental health. For example, a study conducted by Li et al showed that working >60 hours per week is significantly related to poor mental health (OR: 1.66).^40^ Additionally, a previous study based on the EWCS has observed that working >48 hours per week is directly related to poor mental health (OR 1.46).^41^ One of the meaningful findings of our study, which distinguishes it from previous research, is the identification of a dose-response pattern in the relationship between long working hours and the likelihoods of poor mental health. Our observations showed that, as working hours increased, an OR for experiencing poor mental health increased. These findings have practical implications for South Korea’s working hour policy. Unlike European Union countries, which impose limitations on labor exceeding 48 hours per week, South Korea currently adheres to a 52-hour workweek system.^5^ However, our findings indicate that the detrimental effects of long working hours on mental health may emerge even at exposure levels below 52 hours per week.

Our study demonstrated that WLI partially mediated the association of working hours with mental health. This is consistent with prior studies maintaining that long working hours can lead to disturbance of work-life balance.^17^^–^^19^ Long working hours can result in an increase in subjective WLI not only by directly leading to a scarcity of time for socializing with family and friends (time-related WLI) but also by causing fatigue or exhaustion in workers, thereby reducing the energy available for social interactions or family responsibilities (strain-related WLI). Indeed, a recent meta-analysis has demonstrated that long working hours can induce exhaustion, which in turn contributes to WLI.^42^ Previous studies have shown that WLI is a major antecedent of poor mental health.^24^^–^^26^ A sense of disharmony that arises from the inability to effectively manage the demands posed by both work and family roles can contribute to the deterioration of mental health.^43^^,^^44^

In addition to WLI, various other mechanisms may be involved in the association between long working hours and poor mental well-being.^8^ First, a previous study demonstrated that insufficient sleep and irregular mealtimes could act as mediators in the association between working hours and mental health conditions.^45^ Second, elevated occupational stress caused by long working hours can potentially contribute to poor mental well-being.^46^ Third, from a physiological perspective, long working hours may cause a dysregulation of the hypothalamic-pituitary-adrenal axis, potentially leading to the development of mental health disorders.^47^^,^^48^ Interestingly, we observed an increase in the indirect effect as working hours increased, but its proportion in the total effect diminished. This suggests that the effects of long working hours on poor mental health, mediated through an independent mechanism with WLI, increase with the working hours.

Our study has several limitations. First, our analysis used a cross-sectional design. Although we employed the causal mediation terminology of “effect” to improve clarity, a true causal relationships among long working hours, WLI, and poor mental health could not be asserted. For instance, we could not rule out the possibility of reverse causation, in which poor mental health may induce WLI. Additionally, we could not rule out the effect of unmeasured confounders, such as prior psychiatric history, which may lead to biased estimations. Second, our data contained a considerable number of missing values, potentially introducing bias into our estimations. While a complete-case analysis corroborated the mediating role of WLI, we acknowledge that the estimations derived from multiple imputations relied on the MAR assumption, and a potential bias could arise if this assumption is not met. Third, the sixth KWCS was conducted during the coronavirus disease 2019 pandemic, during which the characteristics of working time schedules and WLI significantly changed. Hence, future studies should validate the findings of the present study. Fourth, several sensitivity analyses were conducted to explore whether the association between working hours, WLI, and poor mental health can differ by various workers’ characteristics. However, a mediating effect of long working hours was consistently observed across the gender, occupation, or household composition of workers. Nevertheless, there is a limitation in that factors such as job-demand and control, household chore distribution, and dual-income status were not accurately assessed in these subgroup analyses. Considering that these factors can significantly influence the relationship between working hours, WLI, and mental health, future in-depth studies should explore how this association manifests differently based on workers’ characteristics.

### Conclusion

Our analysis demonstrates the mediating role of WLI in the association of long work hours with poor mental health. Working >40 hours per week was related to an elevated risk of poor mental health, and the disturbance of work-life balance explained 27.6–50.8% of this association. Although longitudinal studies are needed to clarify the causal impacts of long working hours on WLI, and poor mental health, our findings emphasize the necessity for appropriate policies to reduce the adverse impacts of long working hours and overwork on work-life balance.

## SUPPLEMENTARY MATERIAL

eMaterial 1. Factor analysis of WLI scale

eMaterial 2. Counterfactual-based mediation analysis

eTable 1. Items of work-life imbalance scale

eTable 2. Exploratory factor analysis

eTable 3. Confirmatory factor analysis

eTable 4. Results of the sensitivity analysis based on the complete cases (N = 31,418)

eTable 5. Results of the sensitivity analysis based on a different working hour categorization

eTable 6. Results of the sensitivity analysis stratified by gender

eTable 7. Results of the sensitivity analysis stratified by occupation type

eTable 8. Results of the sensitivity analysis stratified by number of household members

eFigure 1. Flowchart of the selection process of study sample
