# Supplementary material for: Long Working Hours, Work-life Imbalance, and Poor Mental Health: A Cross-sectional Mediation Analysis Based on the Sixth Korean Working Conditions Survey, 2020–2021
Source: J Epidemiol. 2024 Nov 5;34(11):535–42. doi: 10.2188/jea.JE20230302 (PMC11464851; doi:10.2188/jea.JE20230302)
Supplement: Supplementary file 1 [file je-34-535-s001.pdf]

eMaterial 1. Factor analysis of WLI scale

The dataset used for factor analysis comprised complete cases (N=31,418), which were evenly partitioned into two groups, one for exploratory factor analysis and the other for confirmatory factor analysis. During the exploratory factor analysis phase, eigenvalues were computed, uncovering a single factor with an eigenvalue exceeding 1 (Eigenvalue=5.03). Subsequently, exploratory factor analysis demonstrated that all factor loadings were 0.5 or greater, jointly accounting for 55% of the total variance (see eTable 3).

eMaterial 2. Counterfactual-based mediation analysis

The causal mediation analysis decomposed the total effect into direct effect (long working hours [x] → poor mental health [y]) and indirect effect (long working hours [x] → WLI score [z] → poor poor mental health [y]). The decomposition of the total effect can be estimated by the following equation[1]:

$$\text{In}\left(\frac{O_{x=1,z|x=1}}{O_{x=0,z|x=0}}\right)[\text{Total effect}] = \text{In}\left(\frac{O_{x=0,z|x=1}}{O_{x=0,z|x=0}}\right)[\text{Indirect effect}] + \text{In}\left(\frac{O_{x=1,z|x=1}}{O_{x=0,z|x=1}}\right)[\text{Direct effect}]$$

In the above equation, the odds of having a poor mental health is presented as O. The left-hand side of the equation (total effect) denotes the odds when the working hour, denoted as x, takes on a specific value (x=1), divided by the odds when x represents the reference exposure level (35–40 hours per week) (x=0). The division is conditioned on the distribution of the mediator (WLI score) in each working hour group ($z|x = 1$ or $z|x = 0$).

The first term on the right side of the equation corresponds to the odds under a counterfactual situation where x is the reference group but assumes the mediator distribution of the specific working hour group ($O_{x=0,z|x=1}$). This term is divided by the odds when x represents the reference group, given the distribution of the mediator of the reference exposure level ($O_{x=0,z|x=0}$). Therefore, it quantifies the indirect effect, which can be interpreted as the effect of long working hours on poor mental health transmitted through high WLI score.

The second term on the right side of the equation represents the odds when x represents the specific working hour group, given the distribution of the mediator of the specific working hour group ($O_{x=1,z|x=1}$). This term is divided by the odds in a counterfactual situation where x is the reference group but assumes the mediator distribution of the specific working hour group ($O_{x=0,z|x=1}$). Therefore, it quantifies the direct effect, which can be interpreted as the effect of long working hours on poor mental health, transmitted through all other potential mechanisms.

---

1 Buis ML. Direct and indirect effects in a logit model. Stata J. 2010 Winter;10(1):11-29.

eTable 1. Items of work-life imbalance scale

Question	Items	Response
	How often in the last 12 months (or since you started your job), have you…?	
**Q1**	kept worrying about work when you were not working	5-point Likert scale (1: "Never" to 5: "Always")
**Q2**	felt too tired after work to do some of the household jobs which need to be done	5-point Likert scale (1: "Never" to 5: "Always")
**Q3**	found that your job prevented you from giving the time you wanted to your family	5-point Likert scale (1: "Never" to 5: "Always")
**Q4**	found it difficult to concentrate on your job because of your family responsibilities	5-point Likert scale (1: "Never" to 5: "Always")
**Q5**	found that your family responsibilities prevented you from giving the time you should to your job	5-point Likert scale (1: "Never" to 5: "Always")
	**Total score**	Range: 5 to 25

eTable 2. Exploratory factor analysis

**Items**	**Loading**
Q1: worriedness	0.57
Q2: Tiredness	0.75
Q3: Time: family	0.75
Q4: Concentration	0.81
Q5: Time: work	0.80

The confirmatory factor analysis utilized a structural equation model was chosen. In the initial model, no correlations between items were assumed. Items Q2 and Q3 were designated to assess work-to-family conflict, while items Q4 and Q5 were utilized to measure family-to-work conflict. In the final model, therefor, the correlation between these items was permitted, aligning with their conceptual similarity. Notably, the model exhibited a good fit, as presented in the results presented in eTable 4.

eTable 3. Confirmatory factor analysis

	**$\chi 2$ [df], *P*-value**	**CFI**	**TLI**	**RMSEA (90% CI)**	**SRMR**
Initial model	7465.734 [5]; p<0.001	0.973	0.945	0.308 (0.302–0.314)	0.088
Final model	226.444 [5]; p<0.001	0.999	0.997	0.069 (0.061–0.77)	0.010

CFI, comparative fit index; RMSEA, root mean square error of approximation; SRMR, standardized root mean square residual ; TLI, Tucker-Lewis index.
To account for the ordered categorical nature of the variables, the Weighted Least Squares Mean and Variance-Adjusted estimator was used.

e**Table 4.** Results of the sensitivity analysis based on the complete cases (N=31,418)

	**Total effect**		**Indirect effect**		**Direct effect**		**PM**
	**OR**	**95% CI**	**OR**	**95% CI**	**OR**	**95% CI**	**%**
**Working hours per week**							
<40 hours	1.00	reference	1.00	reference	1.00	reference	
41–48 hours	1.10	1.02–1.19	1.04	1.03–1.05	1.05	0.97–1.14	44.7
49–54 hours	1.34	1.23–1.47	1.08	1.06–1.10	1.24	1.14–1.35	26.8
≥55 hours	1.72	1.57–1.89	1.14	1.12–1.16	1.51	1.38–1.66	23.8

CI, confidence interval; OR, odds ratio; PM, proportion mediated.

e**Table 5.** Results of the sensitivity analysis based on a different working hour categorization

	**Total effect**		**Indirect effect**		**Direct effect**		**PM**
	**OR**	**95% CI**	**OR**	**95% CI**	**OR**	**95% CI**	**%**
**Working hours per week**							
35–40 hours	1.00	reference	1.00	reference	1.00	reference	
41–46 hours	1.04	0.94–1.14	1.05	1.04–1.07	0.98	0.89–1.09	142.0
47–52 hours	1.19	1.11–1.28	1.05	1.04–1.06	1.13	1.05–1.22	29.2
≥53 hours	1.52	1.41–1.64	1.13	1.11–1.15	1.35	1.25–1.45	28.9

CI, confidence interval; OR, odds ratio; PM, proportion mediated.

eTable 6. Results of the sensitivity analysis stratified by gender

	Total effect		Indirect effect		Direct effect		PM
	**OR**	**95% CI**	**OR**	**95% CI**	**OR**	**95% CI**	**%**
**Working hours per week (Men)**							
<40 hours	1.00	reference	1.00	reference	1.00	reference	
41–48 hours	1.13	1.02–1.25	1.04	1.03–1.06	1.09	0.98–1.20	34.4
49–54 hours	1.38	1.23–1.54	1.08	1.06–1.10	1.27	1.14–1.42	27.6
≥55 hours	1.55	1.39–1.72	1.15	1.12–1.17	1.35	1.21–1.50	31.5
**Working hours per week (Women)**							
<40 hours	1.00	reference	1.00	reference	1.00	reference	
41–48 hours	1.03	0.93–1.14	1.04	1.03–1.05	0.99	0.90–1.09	139.0
49–54 hours	1.16	1.02–1.33	1.08	1.06–1.10	1.07	0.94–1.23	53.0
≥55 hours	1.66	1.47–1.89	1.13	1.11–1.16	1.47	1.30–1.66	24.6

CI, confidence interval; OR, odds ratio; PM, proportion mediated.

eTable 7. Results of the sensitivity analysis stratified by occupation type

	Total effect		Indirect effect		Direct effect		PM
	**OR**	**95% CI**	**OR**	**95% CI**	**OR**	**95% CI**	**%**
**Working hours per week (White collar)**							
<40 hours	1.00	reference	1.00	reference	1.00	reference	
41–48 hours	1.16	1.04–1.30	1.06	1.04–1.08	1.10	0.98–1.23	38.9
49–54 hours	1.79	1.53–2.09	1.11	1.08–1.14	1.61	1.38–1.87	18.2
≥55 hours	1.85	1.50–2.28	1.21	1.16–1.26	1.53	1.24–1.88	31.1
**Working hours per week (non-white collar)**							
<40 hours	1.00	reference	1.00	reference	1.00	reference	
41–48 hours	1.05	0.96–1.15	1.03	1.02–104	1.02	0.93–1.11	66.4
49–54 hours	1.16	1.04–1.28	1.07	1.05–1.08	1.09	0.98–1.20	43.9
≥55 hours	1.58	1.44–1.74	1.11	1.09–1.13	1.42	1.29–1.57	23.1

CI, confidence interval; OR, odds ratio; PM, proportion mediated.

eTable 8. Results of the sensitivity analysis stratified by number of household members

	Total effect		Indirect effect		Direct effect		PM
	**OR**	**95% CI**	**OR**	**95% CI**	**OR**	**95% CI**	**%**
**Working hours per week (number of household member: single)**							
<40 hours	1.00	reference	1.00	reference	1.00	reference	
41–48 hours	1.10	0.94–1.29	1.03	1.01–1.05	1.07	0.92–1.25	30.8
49–54 hours	1.17	0.97–1.42	1.06	1.03–1.09	1.10	0.91–1.34	37.6
≥55 hours	1.29	1.07–1.55	1.11	1.07–1.14	1.16	0.97–1.40	40.2
**Working hours per week (number of household member: non-single)**							
<40 hours	1.00	reference	1.00	reference	1.00	reference	
41–48 hours	1.07	0.99–1.16	1.04	1.03–1.05	1.03	0.95–1.11	61.2
49–54 hours	1.31	1.19–1.45	1.09	1.07–1.10	1.21	1.10–1.33	30.2
≥55 hours	1.69	1.54–1.86	1.15	1.13–1.17	1.48	1.35–1.62	26.1

CI, confidence interval; OR, odds ratio; PM, proportion mediated.

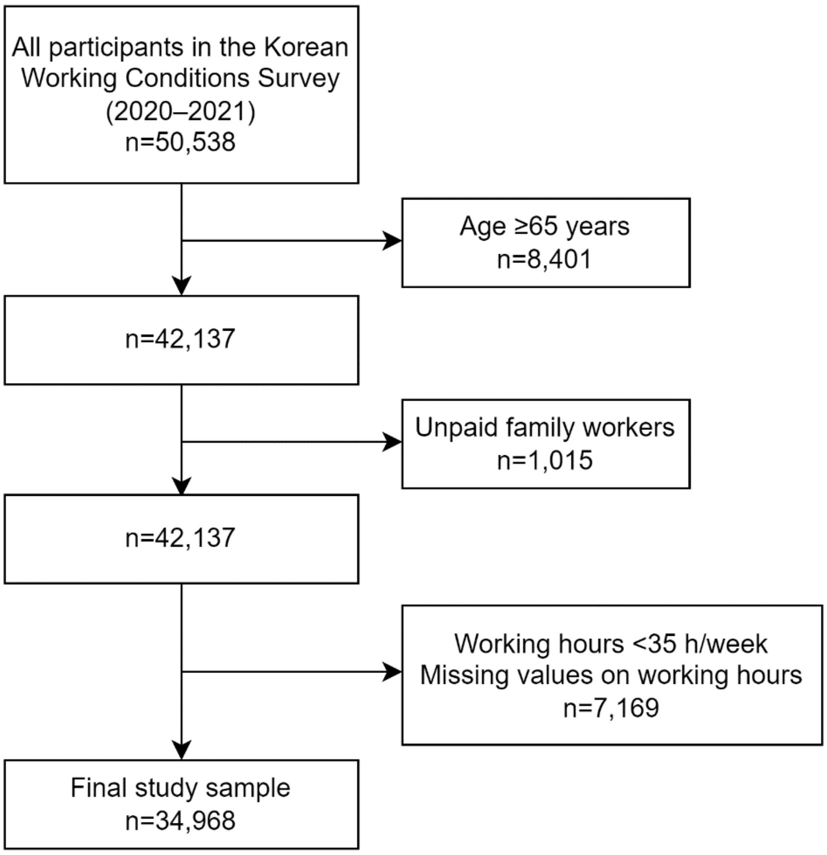

eFigure 1. Flowchart of the selection process of study sample
